# Regulation of Central Nervous System Myelination in Higher Brain Functions

**DOI:** 10.1155/2018/6436453

**Published:** 2018-03-05

**Authors:** Mara Nickel, Chen Gu

**Affiliations:** Department of Biological Chemistry and Pharmacology, The Ohio State University, Columbus, OH 43210, USA

## Abstract

The hippocampus and the prefrontal cortex are interconnected brain regions, playing central roles in higher brain functions, including learning and memory, planning complex cognitive behavior, and moderating social behavior. The axons in these regions continue to be myelinated into adulthood in humans, which coincides with maturation of personality and decision-making. Myelin consists of dense layers of lipid membranes wrapping around the axons to provide electrical insulation and trophic support and can profoundly affect neural circuit computation. Recent studies have revealed that long-lasting changes of myelination can be induced in these brain regions by experience, such as social isolation, stress, and alcohol abuse, as well as by neurological and psychiatric abnormalities. However, the mechanism and function of these changes remain poorly understood. Myelin regulation represents a new form of neural plasticity. Some progress has been made to provide new mechanistic insights into activity-independent and activity-dependent regulations of myelination in different experimental systems. More extensive investigations are needed in this important but underexplored research field, in order to shed light on how higher brain functions and myelination interplay in the hippocampus and prefrontal cortex.

## 1. Structure and Function of the Hippocampus and Prefrontal Cortex

To support rapid encoding of new information and consolidation and organization of memory networks, the brain relies on two important structures, the hippocampus and the prefrontal cortex (PFC). The hippocampus belongs to the limbic system and is a paired structure with mirror-image halves in the left and right sides of the brain. The hippocampus forms and organizes memories, allowing for efficient consolidation of objects, locations, behaviors, and temporal organization at specific events and retrieval of this information at later dates. Hippocampal neurons carry out these functions by communicating with regions of the cerebral cortex. One important region of the cerebral cortex involved in this process is the PFC. The PFC is the anterior part of the cerebral cortex in the frontal lobe. It is responsible for regulating social and cognitive behavior, planning, and decision-making. Neural communications between the PFC and the limbic system lead to behaviors modulated by emotions and motivations. Specific interactions between the medial PFC (mPFC) and the hippocampus orchestrate efficient encoding and retrieval of information to assist in environment-specific actions [1]. While it was thought that the hippocampus was solely responsible for storing new memories, gradually transferring these to the PFC overtime to form remote memory (see review [2]), a recent study has revealed that following initial exposure to a context, both the hippocampus and PFC rapidly form memory cells [3]. Whereas the prefrontal engram cells, with support from hippocampal memory engram cells, become functionally mature with time, hippocampal engram cells gradually became silent. The slow maturation allows the PFC to participate in remote recall, where it may still use the now-silent memory cells of the hippocampus to enhance the recalled memories [3].

Proper communications between the hippocampus and PFC are essential for memory and cognition. The pathway from the hippocampus to the mPFC supports memory consolidation, likely through strong synchronization of their neuronal activities [4–6]. Conversely, the PFC controls memory retrieval processes. Patients with PFC damage displayed deficits under conditions of memory interference or distraction [7]. In animal studies, when the mPFC was temporarily inactivated through infusions of a GABA_A_ receptor agonist, muscimol, rats that were previously trained to employ spatial-contextual rules to guide object selections had decreased task performance [8]. Inactivation of the mPFC actually disrupted the firing pattern of hippocampal neurons during these tasks [8]. Abnormal connections between the hippocampus and the PFC are present in a variety of neurological disorders with cognitive deficits, including Alzheimer's disease, schizophrenia, major depressive disorder, and posttraumatic stress disorder (PTSD) [9, 10]. These abnormalities are thought to dampen the individuals' ability to make appropriate responses to events that cause stress, fear, and so forth [11]. Moreover, cognitive dysfunction was found in more than 50% of multiple sclerosis (MS) patients [12]. Communications between the PFC and hippocampus are disturbed in some patients of MS even before their spatial memory is impaired [13].

Myelinated axons allow rapid and reliable propagation of action potentials over long distances in the nervous system. Demyelination of the axons that connect two separate brain regions can disrupt the communications between them. Myelin alterations in the hippocampus and the PFC were commonly reported in the above-mentioned disorders [14–16]. It is possible that abnormal myelination in the white and gray matter disrupts the unique interaction between the hippocampus and PFC. In fact, this type of disconnection caused by demyelination was shown in the cuprizone mouse model for MS [17]. In this model, ingestion of cuprizone, a copper chelator, leads to apoptosis of myelin-forming cells, thereby causing demyelination in the brain. After twelve weeks of cuprizone treatment, connections between brain regions, especially those involving the hippocampus, were compromised [17]. Myelin is critical for ensuring proper connections throughout the CNS, and dysregulation of myelination may play a key role in the hippocampus-PFC malfunction in many diseases. Limited progress has been made to reveal the mechanisms underlying alterations of myelination in these brain regions, as well as their relevant physiological or pathological significance.

## 2. Myelin Alterations and Related Functions in the Brain

Myelin is composed of compacted lipid membranes that wrap around the axons of many neurons, providing electrical insulation and trophic support. Myelin allows action potentials to propagate along an axon in a saltatory fashion with higher speed and less energy consumption. Whereas Schwann cells are myelinating glia in the peripheral nervous system (PNS), myelin in the central nervous system (CNS) is formed from oligodendrocyte progenitor cells (OPCs) that differentiate into oligodendrocytes (OLs) and form myelin sheaths surrounding axons. Bundles of myelinated axons give rise to the appearance of the white matter. The mechanism and function of myelin in the white matter have been extensively studied. However, many axons in the gray matter that contains neuronal cell bodies and dendrites are also myelinated. A recent study showed that a large fraction of neocortical myelin ensheathes axons of local inhibitory neurons [18]. Gray matter myelination is much less understood and may be regulated differently due to the distinct microenvironments of the white and gray matter.

Myelination is important in establishing connectivity in the growing brain by facilitating rapid and synchronized information transfer across the nervous system, which is essential to higher-order cognitive functions. Once thought of as solely a passive insulator, myelin alteration is now known to be actively involved in the function and development of the CNS (see review [19]). Disruption of myelin can lead to the dysregulation of various neural circuits and give rise to disease symptoms. Uncovering the regulators of myelination has become increasingly important for the diagnosis and treatment of these diseases.

## 3. Regulation of Myelin as a Novel Form of Brain Plasticity

Myelination is not merely a transient event at the perinatal stage. In fact, remodeling of myelin continues throughout adulthood and includes different modes, such as remodeling of existing myelin and new myelination of partially myelinated or unmyelinated axons. Quantification of OPCs and OLs in regions of the adult mouse brain shows that some OPCs are still dividing, differentiating into OLs, and generating myelin [20–22]. In adult human brains, the turnover of OLs may be not as big as that in adult mouse brains, with about 2.5% of all OLs newly added in the adult human cortex (gray matter) annually, but only about 0.33% in the corpus callosum (white matter) [23]. With continued myelin remodeling throughout the lifetime of individuals, neural networks become fine-tuned, contributing to plasticity in the brain (see review [24]). Emerging research has implicated experience- and environment-dependent regulation of myelination in this plasticity. Using diffusion tensor imaging (DTI) in humans, Scholz et al. detected a localized increase in fractional anisotropy, a measurement of microstructure, in the white matter underlying the intraparietal sulcus following training in a complex visuo-motor skill [25]. This result provides evidence of training-related changes in the white matter structure of the healthy human adult brain, which likely reflects altered white matter myelination [25]. Learning-induced change of myelination is also supported by animal studies. Motor skill learning was accompanied by enhanced production of OLs in the mouse brain, whereas blocking new production of OLs inhibited the motor learning [26]. Axonal functions may be altered not only via the changes of the degree of insulation provided by myelin but also via the changes of subcellular domains in myelinated axons. Recent studies showed that geometry changes of nodes of Ranvier and internodes can regulate action potential timing and propagation speed [27, 28].

The hippocampus and the PFC are two brain regions that display significant myelin plasticity in response to experience and environmental cues. Using magnetic resonance imaging (MRI), the Hofstetter group showed that short-term learning can cause significant alterations of the white matter in the fornix of the hippocampus [29]. However, the mechanism and function of experience-related myelin regulation in these two brain regions remain largely unexplored. This represents an emerging and important research field.

Myelination can be regulated in different ways to impact the computation of neural circuits. Different steps of proliferation and differentiation of OPCs and OLs can be regulated to alter the amount of myelin generated, which will be discussed in more detail later. These steps could be altered due to signals from the cells' microenvironment or signals from individual axons. Interestingly, even the pattern of myelin segments along a single axon may be manipulated to alter axonal functions. A recent study revealed distinct profiles of myelin distribution along individual axons of pyramidal neurons in the neocortex [30]. These results suggest that the profile of longitudinal distribution of myelin is an integral feature of neuronal identity and may have evolved as a strategy to modulate long-distance communication in the neocortex. In complex communications such as the one between the mPFC and the hippocampus, myelin alterations may play a key role in the regulation of related higher brain functions.

## 4. Myelination of the Hippocampus during Development and Its Disruption in Diseases

The hippocampus, in coordination with other brain regions, plays an important role in the consolidation of information from short-term memory to long-term memory, as well as in spatial learning and navigation. In humans, the hippocampus is located subcortically in the medial temporal lobe. The hippocampus receives inputs from various cortical and subcortical structures via the perforant pathway from the entorhinal cortex. Information flow in the hippocampus is mainly unidirectional and involves three synaptic connections. Most axons in the perforant pathway project to the granular layer in the dentate gyrus (1st synaptic connection). The dentate granule cell axons (mossy fiber) pass the information to the dendrites of CA3 pyramidal cells (2nd synaptic connection). The mossy fibers are the only axonal tracts exclusively containing unmyelinated axons. From there, CA3 axons (Schaffer collaterals) leave the deep part of the cell body and loop up to the apical dendrites extending to CA1. Axons from CA1 then project back to the entorhinal cortex (3rd synaptic connection) completing the trisynaptic neural circuit. Additional output axons go to other cortical areas including the PFC. There are also interneurons within the hippocampal neural circuits. Myelin can form along the axons projecting into, out of, or within the hippocampus.

**Figure 1:**
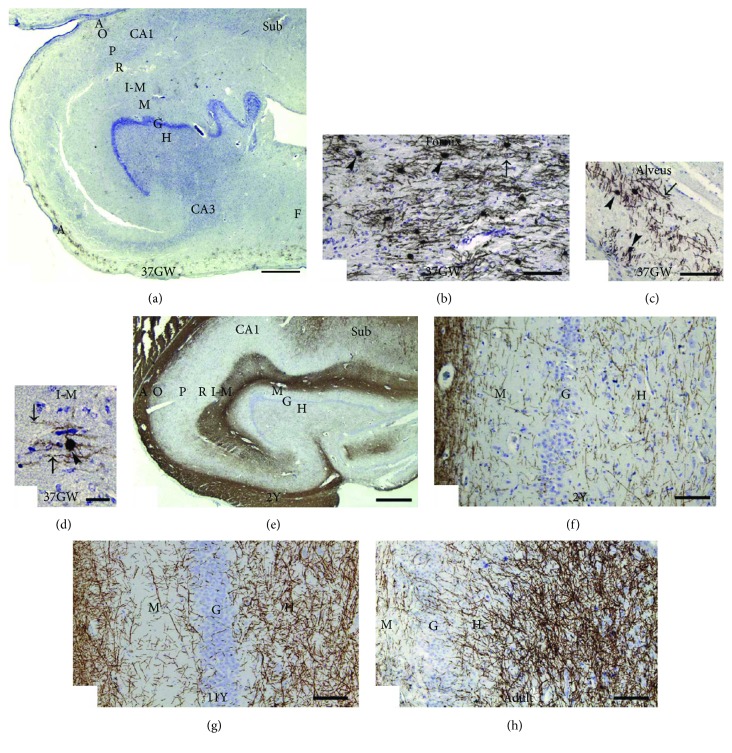
Myelin formation in the hippocampus of humans at different developmental stages. (a) At the 37th gestational week (GW), MBP-positive myelin segments (brown) are present in the fimbria (F), in the entire alveus (A), and in the stratum lacunosum-moleculare (L-M) of the CA1 region. MBP-positive OL cell bodies (arrowheads) and myelin segments (arrows) are present in the fimbria fornicis (b), in the alveus (c), and in the stratum lacunosum-moleculare (L-M) (d) in an infant born at the 37th GW. (e) At 2 years of age, strong MBP staining was observed in the alveus, in the stratum lacunosum-moleculare (L-M) of Ammon's horn, and in the outer half of the molecular layer (M) of the dentate gyrus. MBP-positive myelin segments are also present in the strata oriens (O), pyramidale (P), and radiatum (R) of Ammon's horn. (f) MBP-positive myelin segments in the hilus (H) of dentate gyrus at 2 years of age. (g) MBP-positive myelin segments start to form a dense network in the hilus of the dentate gyrus at the age of 11 years. (h) Much denser network of myelin segments in the hilus of a 53-year-old adult. Scale bars: 1000 *μ*m in (e), 500 *μ*m in (a), 250 *μ*m in (g) and (h), and 100 *μ*m in (b)–(d) and (f). This figure is modified from Abrahám et al. [31], with copyright permission for reusing the figure panels.

Myelination in the limbic system, including the hippocampus and amygdala, is quite complex and has not been extensively investigated. In humans, hippocampal myelination begins during fetal development and continues into postnatal development [31]. Expression of myelin basic protein (MBP), produced by mature OLs, begins around the 20th gestational week in the fimbria fornicis and alveus of human fetal hippocampi (Figure 1) [31]. Postnatally, myelination gradually increases with age, with some regions myelinating more quickly than others [31]. The first myelinated axons appear to be those into and/or out of long-projecting pyramidal neurons. Even into young adulthood, hippocampal myelin continues to increase in volume, exemplifying the early, but prolonged, development of myelin in the hippocampus [31]. Gradual myelination might be important for the prolonged functional maturation of hippocampal circuitry [31]. The continued development of myelin in the hippocampus correlates with functional maturation of the hippocampus into the fifth decade in humans.

Disruption of this progressive myelination is evident in a variety of disorders. It was reported that 53–79% postmortem MS brains showed demyelination in the hippocampus [32, 33]. To reveal the mechanism underlying demyelination, the Dutta group analyzed the morphological and molecular changes of MS hippocampi [34]. Decreased expression of KIF1A motor proteins, presynaptic proteins, glutamate receptors, the glutamate transporter EAAT1 and 2, and CaM kinase II was noted in MS brains compared to those of controls [34]. These proteins are involved in axonal transport, learning and memory, synaptic plasticity, and neuronal survival, which may be responsible for the cognitive deficits seen in more than half of MS patients. Delayed hippocampal myelination is present in individuals with Down syndrome [35]. Patients with Down syndrome often have a below-average IQ due to impairments of cognitive functions. Many of these deficits are hippocampus-dependent. A recent study of developmental gene expression identified defects in OL differentiation and white matter development in Down syndrome brain and in its mouse model, providing a transcriptional framework for investigating Down syndrome pathogenesis [36].

Moreover, demyelination in the hippocampus was observed in patients with Alzheimer's disease, temporal lobe epilepsy, or psychotic disorders [34, 37–39]. Interestingly, social isolation has been associated with demyelination in the hippocampus, similar to what is seen in Alzheimer's patients. A recent study showed that 17-month-old mice housed alone for 3 months showed impaired learning and memory, in concurrence with a decrease in hippocampal volume and myelin-associated protein expression, a signature of Alzheimer's disease [40]. While the reduced volume of the hippocampus is a confounding variable for decreased myelin gene expression, in the mouse model of Alzheimer's disease, the myelin segments remaining in the hippocampus are shorter, especially in the dentate gyrus, which could contribute to the memory impairments of the mice [14].

Abnormal conditions are also associated with increased myelin in the hippocampus. A recent study focusing specifically on the hippocampus indicated that veterans with PTSD had significantly increased hippocampal myelin compared to trauma-exposed controls [16]. It appeared that hippocampal myelination positively correlated with depressive symptom severity in PTSD [16]. The notion is supported by the study in mice, in which it was shown that stress-related glucocorticoids can promote myelination in the adult hippocampus [41]. Taken together, these findings indicate the importance of myelin regulation: myelin must be at an optimal level, not too little or too much, to achieve normal functioning of the hippocampal neural circuit. The mechanism and function of myelin regulation in the hippocampus deserve further investigation.

## 5. Myelination in the PFC and Its Alterations by Social Experience and Alcohol Consumption

The PFC is among the last brain regions to mature in humans [42]. It is highly interconnected with many other brain regions, including cortical, subcortical, and brain stem sites. The PFC can be divided into four major subregions, the medial PFC (mPFC), the orbitofrontal cortex, the lateral PFC, and the caudal PFC. In particular, the dorsal PFC is especially interconnected with brain regions involved in attention, cognition, and action, while the ventral PFC interconnects with brain regions involved in emotion. Similar to the hippocampus, myelination of the PFC, as well as of other parts of the human neocortex, continues well into early adulthood [43]. The PFC plays a key role in decision-making in response to situational contexts. While the PFC uses past experiences to make future decisions, the past experience can regulate PFC functions.

**Figure 2:**
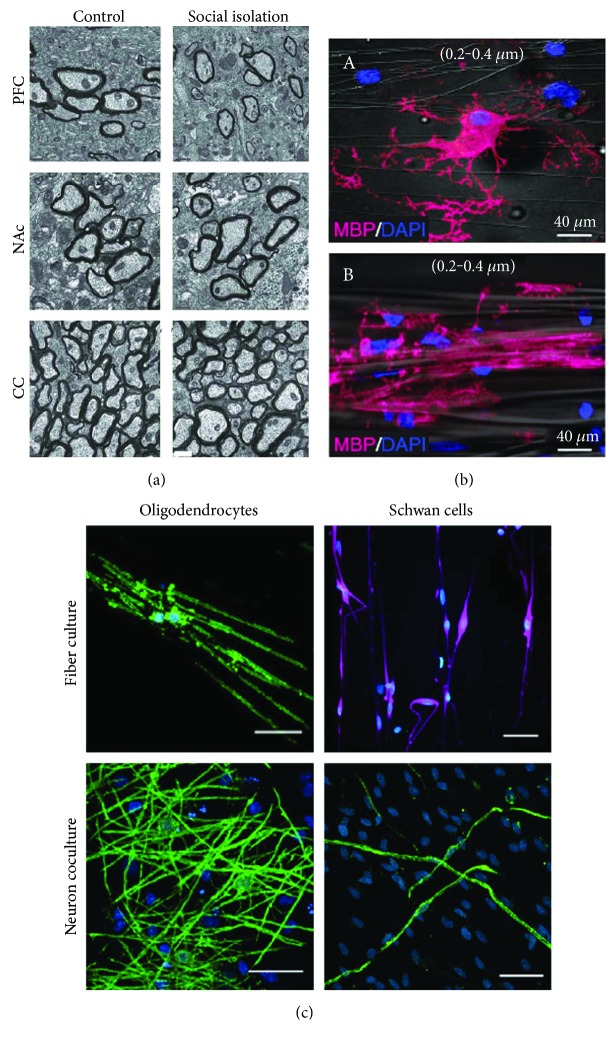
Axonal activity-dependent and activity-independent regulation of myelination. (a) Prolonged social isolation of adult mice induces hypomyelination in the PFC. Electron micrographs of axons in the PFC, nucleus accumbens (NAc), and corpus callosum (CC) from control and isolated mice (modified from Liu et al. [47], with copyright permission for reusing the figure panels). (b) The fiber diameter is sufficient to initiate myelination in a neuron-free culture. To determine the minimum fiber diameter at which oligodendrocytes commence wrapping in our system, Lee et al. analyzed oligodendrocytes cultured on nanofibers ranging from 0.2 to 4.0 *μ*m in diameter and quantified the total number of MBP^+^ segments normalized to the fiber distribution on each coverslip. The minimum fiber diameter threshold for oligodendrocyte myelination is approximately 0.4 *μ*m, which is supported by immunostaining of cultures for MBP and DAPI in the presence of small-diameter (A, 0.2–0.4 *μ*m) and large-diameter (B, 2.0–4.0 *μ*m) fibers. Electron-spun polystyrene or poly-L-lactic acid (PLLA) nanofibers with diameters ranging from 0.2 *μ*m to 4.0 *μ*m were engineered (modified from [62] with copyright permission for reusing the figure panels). (c) OLs have the unique, intrinsic capability to generate compact membrane sheaths and physiological internode lengths on microfibers. Confocal stacks of rat primary cortical OLs or Schwann cells cultured 14 or 21 days, respectively, on 1-2 *μ*m microfibers or neurons (green: MBP, blue: Hoechst, and purple: S-100) (modified from Bechler et al. [64], with copyright permission for reusing the figure panels). Scale bars: 0.5 *μ*m in (a) and 40 *μ*m in (b) and (c).

Social experience has significant and long-lasting effects on myelination in the PFC during early development. Researchers found that the white matter in the mPFC changed in children raised in neglectful institutions among Romanian orphanages and that the changes were irreversible following foster care placement [44, 45]. In concurrence with this finding, the Makinodan group conducted a study in which mice were placed in either an isolated, standard, or enriched environment [46]. While all mice had the same density of mPFC OLs, the mice that experienced social isolation exhibited OLs with simpler morphology and shorter branching, contributing to decreased expression of myelin genes and reduced mPFC myelin thickness (Figure 2(a)). In addition, the mice in social isolation showed decreased social interactions and working memory. These effects were unable to be reversed following reexposure to social interactions. Interestingly, they discovered the influence of social isolation on myelin occurring only during a critical period—isolation longer than this critical period did not lead to a further reduction in myelin, and mice isolated at a time after this critical period showed no difference in OL or myelin morphology compared to those in a regular environment [46].

Environmental alterations during a critical period of myelin maturation can lead to long-lasting cognitive and behavioral deficits. Similar to myelination in adolescence, the ongoing myelination during adulthood can be influenced by environment or experiences, exemplifying its role in brain plasticity, but appears to be reversible following the event [47]. Eight weeks of social isolation was sufficient for adult mice to exhibit signs of social withdraw in a PFC-dependent behavior [47]. These isolated mice showed a significant decrease in expression of OL-specific genes in the PFC. Although axons in the PFC were still myelinated, the myelin sheath was thinner, and there were decreased myelin gene transcripts as well. OLs of the isolated mice displayed increased euchromatin and decreased heterochromatin, indicating a less differentiated state [47]. Accordingly, there were increased markers for acetylation, decreased histone methylation markers, and decreased expression of the enzymes regulating histone acetylation and methylation. Unlike adolescent mice, reintegration of adult mice into a social environment was adequate to return myelin levels and social behavior to normal [47]. To confirm the role of myelination in social isolation, clemastine treatment, which can promote OL differentiation and myelination, enhanced myelination in the PFC and rescued behavioral changes in socially isolated mice [48].

PFC myelination can also be damaged by heavy alcohol consumption and may be involved in the behavioral and cognitive impairments associated with alcoholism. In a rat model, adolescent binge drinking reduced myelin density in the mPFC and disrupted T-maze working memory in adulthood [49]. This finding is consistent with that of a more recent study in which researchers found a correlation between alcohol use severity in humans and lower white matter factor scores [50]. Future investigations are needed to determine the mechanism underlying alcohol-mediated myelin damage in the PFC and throughout the CNS and its relevance to alcohol-related behavioral changes. Taken together, myelin in the PFC likely plays a key role in eliciting PFC-dependent behaviors under normal and abnormal conditions.

## 6. Differential Effects of Experience on Myelination in the Hippocampus and the PFC

Whereas social interactions seem to affect PFC and hippocampal myelin in a similar manner, with isolation resulting in decreased myelination in both regions, other experiences may cause different effects. This was observed in response to stress, a condition studied for its association with many neurological and mental disorders [51]. Mice exposed to a stressor (social defeat, forced swimming, or restraint) once a day for 21 days showed alterations in mPFC-dependent behaviors, such as sociability, working memory, and spatial reference memory [52]. In the mPFC, OPCs with reduced processes, a reduction of mature OLs, and severe hypomyelination were observed. To demonstrate that the stress-related behavioral changes were due to demyelination, Yang et al. induced demyelination using lysophosphatidylcholine injections and found similar cognitive and social deficits [52]. In contrast, a study by Chetty et al. showed that adult rats exposed to immobilization stress displayed decreased neurogenesis and increased OLs from neural stem cells in the dentate gyrus of the hippocampus [41]. Although this study did not examine how increased OL production altered myelin content, it appears consistent with the report that increased hippocampal myelin and decreased PFC white matter were found in PTSD, a disease in which stress is the ultimate cause [16].

The opposite effects of social isolation and stress on myelination in the PFC and the hippocampus could contribute to the behavioral and cognitive differences between diseases such as Alzheimer's disease and PTSD. While both diseases involve neural disconnections between the PFC and hippocampus, recent studies showed that increased myelin in the hippocampus and decreased white matter in the PFC observed in PTSD may lead to stronger connections between the hippocampus and the amygdala, causing heightened fear responses that cannot be modulated by the PFC [53–55].

Experience-dependent regulation of myelination in the hippocampus and the PFC deserves further investigation into the underlying mechanisms, due to physiological significance under both normal and abnormal conditions. Some progress has been made in understanding the potential mechanisms underlying intrinsic and extrinsic regulation of myelination using different experimental systems. These findings may shed light on experience-dependent regulation of myelination in the two key regions involved in higher brain functions.

## 7. Regulation of Myelin Formation Independent of Neuronal Activity

Myelin formation in the CNS contains multiple highly regulated steps including proliferation of OPCs and differentiation of OLs. During development, OPCs are generated from the neural stem cells in different regions at different times. These OPCs then proliferate and migrate throughout the CNS, and upon reaching their destination, they differentiate into OLs and myelinate axons. So far, both extrinsic and intrinsic mechanisms have been implicated in the regulation of these steps [56].

The microenvironment of OPCs and OLs, including locally secreted factors, can influence their development, independent of neuronal activity. This may in part explain the varying degree of myelin plasticity between regions in the CNS. Using organotypic slice cultures from the forebrain and cerebellum of early postnatal mice, the Hill group compared basal and platelet-derived growth factor- (PDGF-) induced proliferation of neural/glial antigen 2- (NG2-) positive OPCs in different brain regions [57]. They found that the OPCs in the white matter but not in the gray matter appeared to proliferate in response to PDGF, suggesting intrinsic mechanisms for the differential proliferative response of OPCs [57]. Consistent with this finding, using BrDU labeling and mouse genetics focusing on optic nerves, the Young group found that while all OPCs continue to divide throughout the CNS into adulthood, the rate of division appears greater in the white matter than in the gray matter [58]. They suggested that the optic nerve in adulthood mainly employs the “myelin remodeling” strategy—replacing and restructuring myelin on fully myelinated axons, whereas other brain regions such as the corpus callosum may use “de novo myelination” strategy—through myelinating previously unmyelinated axons [58]. In humans, the degree to which experience regulates the myelin content may vary between regions. While about 75–90 percent of the variation in the white and gray matter of the frontal and temporal lobes is likely controlled by genetic factors, the corpus callosum white matter is more likely to be regulated by environmental changes [59, 60].

Recent studies have refined our view of axon-OL interactions during myelination. It was previously thought that axonal activity is required for OL differentiation and myelination. However, the Rosenberg group showed that packing constraints provided by artificial beads were sufficient to induce OL differentiation [61]. Surprisingly, OPCs seeded onto fixed axons also differentiated and formed compact myelin, clearly indicating that initiation of OL differentiation and myelination did not require dynamic axonal activity [61, 62] (Figure 2(b)). A cell-intrinsic timer was proposed to control OL development, when to divide and when to differentiate [63]. In a study by Bechler et al., region-specific OLs were able to myelinate without axons present and with myelin internode lengths consistent with their specific brain region [64]. The OLs formed initial, multilayered myelin sheaths on microfibers, in a neuron-free culture. Interestingly, Schwann cells, the PNS myelinating glia, were not able to myelinate the microfibers (Figure 2(c)), suggesting that the intrinsic properties are exclusive to the CNS. In addition, when OLs from both the spinal cord and the cortex were isolated with the microfibers, the length of the myelin along the microfibers produced by spinal cord-OLs was significantly longer, consistent with their length in the CNS, suggesting that the intrinsic mechanism is region-specific [64]. Neuronal activity-independent factors can be responsible for some alterations of myelin in the hippocampus and the PFC. However, neuronal and axonal activities have been found to still play important roles in regulating myelin at the levels of OPC proliferation, OL differentiation and maturation, and myelin sheath stabilization.

## 8. Regulation of OPC Proliferation and Differentiation by Neuronal Activity

**Figure 3:**
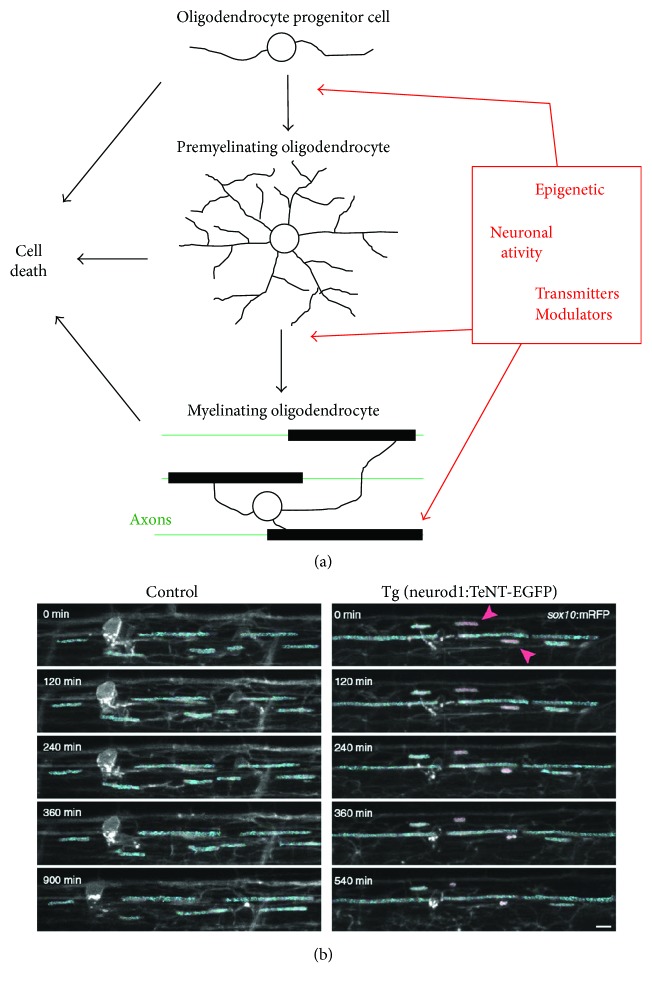
Mechanisms underlying activity-dependent regulation of myelination. (a) Diagram of the multiple steps involved in the development of OLs. OPCs differentiate into multipolar premyelinating OLs, which mature into myelinating OLs. Mature OLs form myelin segments on multiple axons simultaneously. Regulation can occur at different steps during development, eventually leading to altered myelin formation. (b) Nascent myelin sheaths are stabilized by activity-dependent secretion. Representative confocal images show the retraction of existing sheaths during 15 h time-lapse imaging in sibling control (left) and *Tg(neurod1:TeNT-EGFP)* larvae (right). In the right panel, expression of TeNT-EGFP disrupted axonal activity-dependent secretion. Images are lateral views of the dorsal spinal cord, and the time relative to the start of image acquisition is indicated for each image. For demonstrative purposes, sheaths stable for the entire time-lapse are shaded in blue. Retracting sheaths are shaded in red and are also indicated by red arrowheads (modified from Hines et al. [71], with copyright permission for reusing the figure panels). Scale bar: 5 *μ*m.

Neuronal activity can regulate the early steps of myelin formation, including OPC proliferation and differentiation, to impact overall myelination (Figure 3(a)). Several secreted molecules from active neurons or responding glia have been reported to increase OPC proliferation and/or differentiation, including PDGF, brain-derived neurotrophic factor (BDNF), adenosine 5′-triphosphate (ATP), and glutamate (see review [65]). In addition, OPCs express some receptors of neurotransmitters and can be directly regulated by synaptic transmission [66].

Two recent studies showed that activity-dependent regulation of OPCs takes place in motor learning. The first showed that optogenetic stimulation of premotor cortex neurons in awake and behaving mice led to OPC proliferation and increased oligodendrogenesis and myelination, within the deep layers of the premotor cortex and subcortical white matter [67]. This effect was accompanied by improved motor function of the corresponding limb. Inhibiting epigenetic changes that are required for OPC differentiation eliminated this activity-dependent stimulation [67]. This finding appears consistent with that of the second study showing that learning a new motor skill alters the brain's white matter by promoting OPC proliferation and differentiation into OLs [26]. By genetically manipulating the transcription factor in OPC proliferation, researchers specifically blocked the production of new OLs during adulthood without affecting preexisting OLs or myelin. This prevented mice from acquiring a new complex motor skill [26].

Activity-dependent myelination also occurs in sensory inputs. Etxeberria et al. found that manipulation of sensory experience through visual stimuli was able to modulate the myelin in the optic nerve in what seems to be an activity-dependent manner [68]. In this study, mouse models of monocular deprivation showed an increase in mature OLs in the optic tract and optic nerve. Interestingly, neither the number of myelinated axons nor the thickness of myelin sheaths was altered in the optic nerve following monocular deprivation. However, the length of the myelin internodes was decreased, and the number of nodes of Ranvier was increased. These morphological changes were accompanied by a 22.1% reduction in optic nerve action potential conduction velocity [68]. Thus, visual experience impacts axonal activity in optic nerves, which modulates myelin length by altering OPC maturation.

Activity-dependent modulation of OPCs has been implicated in stress-dependent regulation of myelin in the hippocampus and PFC. In stress-promoted oligodendrogenesis in the hippocampus of adult rats, the results were recapitulated when treating the rats with the stress hormone cortisone [41]. The cortisone treatment increased prooligodendrogenic transcription factor expression and decreased inhibitory transcription factor expression in cultured neural stem cells, likely via glucocorticoid receptors on the OPC cell membranes [41]. In contrast, the study of the mPFC showed that chronic stress including social defeat, forced swimming, and restraint in mice led to severe hypomyelination via a negative regulation of OPC proliferation and maturation [52]. Death receptor 6 activation and the caspase 3 pathway were implicated in the OPC reduction [52]. However, in these studies, it is still not clear whether the chemical production of stress, stress-related neuronal activity, or both regulates OPC proliferation and differentiation and ultimately myelin production.

## 9. Regulation of OL Maturation and Myelination by Neuronal Activity

Neuronal activity can regulate myelin formation at the levels of OL differentiation and myelination (Figure 3(a)). A recent study suggested that electrically active axons are preferentially myelinated via interactions between the released vesicles and OLs, leading to OL maturation [69] (Figure 3(a)). Wake et al. used cultures containing both dorsal root ganglion (DRG) neurons treated with clostridial neurotoxin, botulinum A (BoNT/A), a potent enzyme of vesicle fusion at the synaptic membrane, and untreated DRG neurons. When cultured with OPCs, the axons of untreated neurons were preferentially myelinated over those of treated neurons. In addition, when synaptic vesicles were completely blocked and the neurons were electrically stimulated, preferential myelination was still evident, suggesting that synaptic and nonsynaptic vesicles are able to induce OL formation and subsequent myelination [69]. In addition to this study, Mensch et al. used zebrafish to study the myelinating capabilities of individual OLs in response to neuronal activity [70]. Zebrafish were treated with or without tetanus toxin, a treatment that inhibits synaptic vesicle release. They discovered that the number of myelin sheaths produced by individual OLs was reduced by roughly 30% when the synaptic activity was disrupted. In addition, by using a GABA_A_ receptor antagonist, they found that increased synaptic activity led to a roughly 40% increase in myelination [70]. Therefore, the current theory is that while axons with diameters above a certain threshold can be wrapped by myelin membranes, myelin segments are more stable along electrically active axons. Using time-lapse imaging in zebrafish, a recent study by the Hines group showed that nascent myelin sheaths are stabilized by activity-dependent secretion (Figure 3(b)) [71]. Recent studies from two independent groups discovered that activity-dependent Ca^2+^ transients in developing myelin processes regulate sheath elongation, also using zebrafish as the *in vivo* model [72, 73].

Activity-dependent regulation of myelination was implicated in the studies showing that social isolation led to myelin disruption in the mPFC [47, 48]. Reduced myelin in socially isolated mice can be mimicked by disruption of the neuregulin-1/ErbB (NRG1-ErbB) signaling pathway in OLs [46]. Although the recovery of myelin differs between adolescent and adult mice after social isolation, their effect on myelin in the PFC seems to be consistent. It was implicated that the epigenetic changes driven by social isolation during adulthood could influence expression of members of the NRG1-ErbB pathway. Interestingly, using a myelin coculture system, Lundgaard et al. later showed that NRG is sufficient to switch OLs from activity-independent to activity-dependent states by increasing NMDA receptors in OLs, allowing for accelerated and increased myelination in response to glutamate release by neurons [74]. Despite some progress, our understanding of the mechanisms underlying activity-dependent regulation of OL differentiation and myelination is still very limited, especially those in hippocampus and PFC.

## 10. Future Perspectives

Regulation of myelination likely plays a key role in experience-induced long-term alteration of higher brain functions. In particular, myelin regulation in the hippocampus and the PFC may have long-lasting effects on memory, cognition, decision-making, and social behaviors. Importantly, myelination of these two regions involving both the gray and white matter takes decades to fully mature, and myelin plasticity may occur throughout an individual's lifetime. Although some progress has been made, our understanding of activity-independent and activity-dependent regulations of myelination remains very limited in many brain regions, including the hippocampus and the PFC. Moreover, compared to white matter myelination, we currently still know little about gray matter myelination. While synaptic plasticity in the gray matter is an established form of neural plasticity, myelin alteration may be a novel form and remains to be fully understood. However, to further complicate the issue, myelin regulation appears region-dependent. Therefore, to elucidate specific molecular mechanisms governing the regulation of myelin formation in the hippocampus and the PFC, both *in vitro* and *in vivo* experimental systems deserve major efforts in future investigations. Perhaps, it is more important to determine whether there is a unified mechanism underlying activity-dependent regulation of myelination throughout all brain regions. Additionally, the exact physiological and pathological roles of myelin alterations must be better understood. For instance, future research should reveal how abnormal myelination is involved in the pathogenic process in various neurological and psychological disorders and conditions, such as Alzheimer's disease, Parkinson's disease, alcohol abuse, and PTSD. Knowing the specific sites of interruption can guide research in targeted therapies for myelin disruption. The findings of such research will contribute to the development of novel strategies for treating these devastating disorders and conditions.
